# Sigmoid Colon Angiomyolipoma as a Culprit for Intermittent Constipation and Hematochezia

**DOI:** 10.14309/crj.0000000000001502

**Published:** 2024-09-12

**Authors:** Amel Tabet Aoul, Ama Achiamah, Nathaniel Leavitt, Chun He, Pujan Kandal, Varun Patel

**Affiliations:** 1 Department of Internal Medicine, HCA Healthcare Florida Citrus Hospital Florida, Inverness, FL; 2 Department of Pathology, HCA Healthcare Florida Citrus Hospital Florida, Inverness, FL; 3 Division of Gastroenterology, Department of Internal Medicine, HCA Healthcare Florida Citrus Hospital, Florida, FL

**Keywords:** colonic angiomyolipoma, hematochezia, perivascular epithelioid cell neoplasm, colonic semipedunculated polyp

## Abstract

Colonic angiomyolipomas (CA) are very rare benign tumors arising from perivascular epithelioid cells. CA are most often found either during screening colonoscopies or as an incidental finding during abdominal imaging. However, some rare cases of CA are found to present with abdominal pain and hematochezia. In this article, we report a case of a 62-year-old man who presented with intermittent hematochezia and constipation who was found to have an angiomyolipoma in the sigmoid colon. The lesion was successfully removed endoscopically with no recurrence of bleeding and no complications within the first 30 days after the procedure.

## INTRODUCTION

Among the numerous existing mesenchymal tumors, angiomyolipoma, also known in the past as hamartoma, refers to benign tumors composed of variable-sized blood vessels, spindle-cell–type smooth muscle tissue, and mature adipose tissue.^1^ Morgan et al coined the original name in 1951, but in 2002, the World Health Organization classified angiomyolipoma under the umbrella of mesenchymal tumors derived from perivascular epithelioid cells also known as PEComas.^2,3^

Angiomyolipomas are mostly encountered as benign renal tumors, more prevalent in females in their 30s to 40s, and very often associated with tuberous sclerosis.^1^ Angiomyolipomas rarely present extrarenally, but have been found in the head, hard palate, nasal cavity, mediastinum, skin, retroperitoneum, liver, vagina, fallopian tube, penis, and spermatic cord.^4–7^

Colonic angiomyolipomas are extremely rare, and only a few cases have been described in the literature to date. Contrary to their renal counterparts, in previous studies, colonic angiomyolipomas were predominantly found in middle-aged men with no history of tuberous sclerosis. These patients were either asymptomatic and found incidentally during screening colonoscopy or other abdominal imaging or moderately symptomatic with abdominal pain and fullness sensation.^4,8–18^

In this article, we present a rare case of sigmoid colon angiomyolipoma that was diagnosed in a male patient presenting with chronic anemia and intermittent constipation and hematochezia.

## CASE REPORT

**Figure 1.**
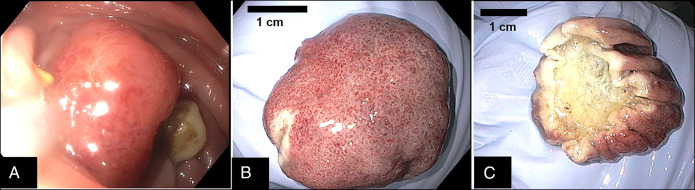
Colonic angiomyolipoma: (A) endoscopic view before resection, (B) external mucosal view, and (C) flipped mass to show resection side.

**Figure 2.**
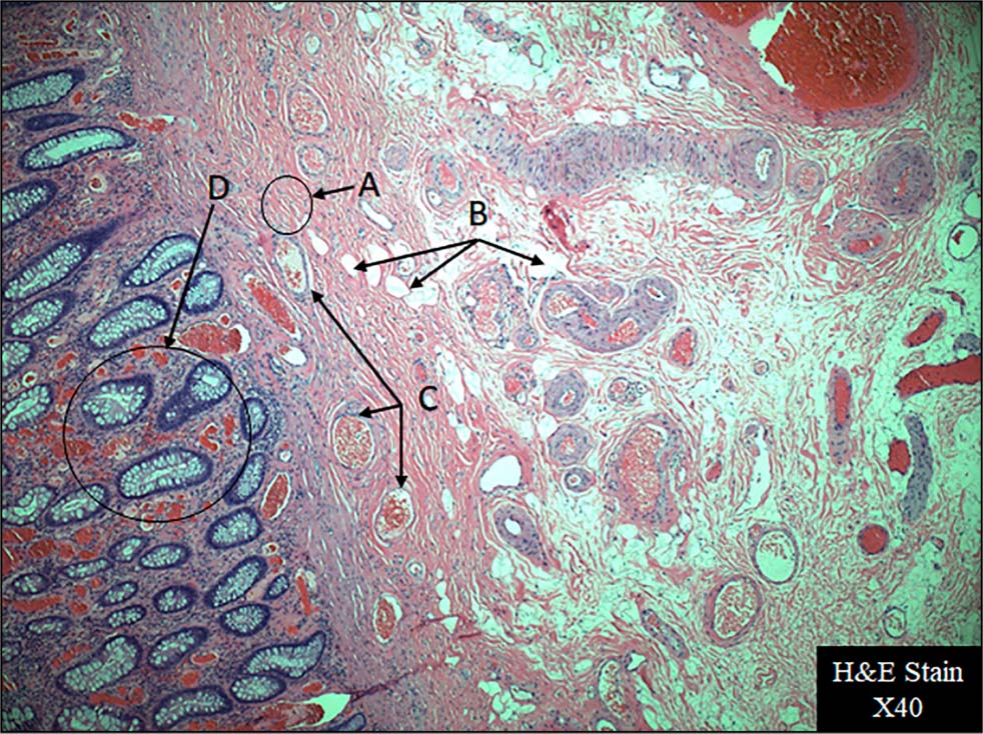
Histopathology of sigmoid colon angiomyolipoma: Arrow (A) shows smooth muscle tissue (spindle cell type), arrow (B) shows fat tissue, arrow (C) shows variable-sized blood vessels, and arrow (D) shows hyperplastic colon mucosa. H&E, hematoxylin and eosin.

Our patient was a 62-year-old man with a nonsignificant medical history who presented to our gastroenterology clinic in mid-2023. He had a new onset of anemia and complaints of intermittent constipation and hematochezia for the past 7 years with no other alarm symptoms. The patient denied any personal or family history of tuberous sclerosis or gastrointestinal malignancies and had never undergone a colonoscopy. An abdominal/pelvic computed tomography (CT) with contrast CT performed 6 months before his visit to the clinic was remarkable for extensive sigmoid diverticulosis, but no renal or colonic masses were detected. He was otherwise hemodynamically stable, and his blood work completed 2 months earlier was only remarkable for mild normocytic normochromic anemia with a hemoglobin 11.5 g/dL, hematocrit 32.7%, and platelets 321 × 10^9^/L. The patient underwent a colonoscopy that revealed moderate diverticulosis of the sigmoid and descending colon with no evidence of diverticular bleeding. In addition, there was a large (3-cm), semipedunculated polypoid mass in the sigmoid colon that was 23 cm from the anal verge. This polyp was resected through endoloop with a hot snare polypectomy (Figure 2) along with a 5-mm non-neoplastic hyperplastic polyp found in the rectum. The resection of the pedunculated polyp was complete with negative margins, and its histopathology study with hematoxylin and eosin stain at 40× (Figure 1) showed mature adipose tissue, variable-sized blood vessels, and smooth muscle tissue (spindle cell type), along with hyperplastic colon mucosa. The diagnosis of angiomyolipoma was made, and no further histochemical staining was necessary. The patient tolerated the procedure well with no intraoperative bleeding, and he was discharged home the same day. He only experienced very mild hematochezia with defecation over the next 2 days, which resolved completely on its own. Thus, no further CT was performed since total resolution of symptoms and no bleeding in 30 days with a stable hemoglobin level. The patient was then instructed to continue an appropriate maintenance bowel regimen with a fiber-rich diet, laxative, and supplementary fiber to prevent constipation and diverticular complications and to undergo a follow-up colonoscopy in a year.

## DISCUSSION

Because of their histological characteristics and although considered benign tumors, angiomyolipomas can be dangerous and have the propensity to bleed and sometimes to invade locally and even noncontiguous organs.^19^ Several authors have reported hemorrhaging angiomyolipomas, but in most cases, they were either renal or extrarenal retroperitoneal tumors.^19^ In the case of colonic angiomyolipomas, most reported cases presented with abdominal pain or fullness sensation or were completely asymptomatic, and the tumors were only discovered incidentally during a screening or imaging procedure.^4,10^ However, as with our patient, there are 2 previous cases where symptoms at presentation were hematochezia. The first case was described by Baek et al, where a 16-year-old girl with a 2 × 2.5-cm submucosal angiomyolipoma in the transverse colon was resected endoscopically.^11^ The second case was described by Perumal et al the mass involved the ileocecal junction requiring a hemicolectomy with an ileotransverse colon anastomosis.^10^ In our patient, his angiomyolipoma was in the form of a semipedunculated polyp in the sigmoid colon, which presented with both intermittent constipation and hematochezia. Most reported cases of colonic angiomyolipoma had a polypoid and pedunculated shape ranging from 1 to 5 cm and affected mostly the left colon, but only a few were removed endoscopically through hot snare.^4,9,13^ Others, however, required surgical resections either because of the tumor's location or because of its size.^8,14,16,17^ In all reported cases, there was no association with tuberous sclerosis, and only a few physician researchers undertook a histochemical staining of the polyps, given their characteristic microscopic appearance. These researchers found that, in general, although renal angiomyolipomas stained positive for smooth muscle actin and HMB45, the colonic ones stained positive for SMA but very rarely reacted to HMB45.^17–19^ In our patient's case, the histopathologic appearance was very characteristic of a colonic angiomyolipoma, not necessitating any further histochemical staining.

We presented the case of a 62-year-old man diagnosed with colonic angiomyolipoma that manifested as intermittent constipation and rectal bleeding. We believe the large semipedunculated polyp was causing intermittent obstruction. Our patient responded well after endoscopic endoloop-assisted hot snare resection of the polyp. Our thorough review of the literature showed that colonic angiomyolipoma is a very rare type of mesenchymal tumor affecting the gastrointestinal tract. What stood out as even more interesting is that patients were mostly either asymptomatic or complained of abdominal pain and discomfort, whereas only 2 cases to the best of our knowledge presented with hematochezia, as was the case for our patient. Thus, through this case report, we endeavor to increase awareness around a rare etiology for anemia and intermittent bowel obstruction and rectal bleeding that can occur.

## DISCLOSURES

Author contributions: A. Tabet Aoul and P. Kandal contributed equally to literature review, drafting of the manuscript, and approved the final draft submitted. A. Achiamah, N. Leavitt, C. He, and V. Patel revised the manuscript and approved the final draft submitted. P. Kandal is the article guarantor.

Acknowledgment: Michael G. Flynn, PhD, Medical Writer, provided writing and copyediting assistance on the final version of the manuscript.

Financial disclosure: None to report.

Disclaimer: This research was supported (in whole or in part) by HCA Healthcare and/or an HCA Healthcare-affiliated entity. The views expressed in this publication represent those of the author(s) and do not necessarily represent the official views of HCA Healthcare or any of its affiliated entities.

Informed consent was obtained for this case report.
